# Effectors of the Type VI Secretion System Have the Potential to Be Modified into Antimicrobial Peptides

**DOI:** 10.1128/spectrum.00308-23

**Published:** 2023-07-20

**Authors:** Wenjia Lu, Hao Lu, Chenchen Wang, Gaoyan Wang, Wenqi Dong, Chen Tan

**Affiliations:** a National Key Laboratory of Agricultural Microbiology, College of Veterinary Medicine, Huazhong Agricultural University, Wuhan, Hubei, China; b Hubei Hongshan Laboratory, Wuhan, China; c The Cooperative Innovation Center for Sustainable Pig Production, Wuhan, Hubei, China; d Hubei Hongshan Laboratory, College of Veterinary Medicine, Huazhong Agricultural University, Wuhan, Hubei, China; e Key Laboratory of Preventive Veterinary Medicine in Hubei Province, The Cooperative Innovation Center for Sustainable Pig Production, Wuhan, Hubei, China; University of Pennsylvania; University of South Florida

**Keywords:** T6SS effector, antimicrobial peptides, multidrug-resistant, Tsap, anti-inflammation

## Abstract

The use of antibiotics has led to the emergence of multidrug-resistant (MDR) bacteria, and there is an urgent need to find alternative treatments to alleviate this pressure. The type VI secretion system (T6SS) is a protein delivery system present in bacterial cells that secretes effectors that participate in bacterial virulence. Given the potential for the transformation of these effectors into antimicrobial peptides (AMPs), we designed T6SS effectors into AMPs that have a membrane-disrupting effect. These effectors kill bacteria by altering the membrane potential and increasing the intracellular reactive oxygen species (ROS) content. Moreover, AMPs also have a significant therapeutic effect both *in vivo* and *in vitro*. This finding suggests that it is possible to modify bacterial components of bacteria themselves to create compounds that fight bacteria.

**IMPORTANCE** This study first identified and modified the T6SS effector into positively charged alpha-helical peptides. These peptides have good antibacterial and bactericidal effects on G+ bacteria and G− bacteria. This study broadens the source of AMPs and makes T6SS effectors more useful.

## INTRODUCTION

Antibiotic resistance causes difficulties in clinical treatment and results in serious economic losses (1). Antibiotics are vital for treating patients with multidrug-resistant (MDR) bacterial infections (2). From the elucidation of the mechanisms of action of classical first-line drugs to the discovery of the carbapenem resistance gene MCR-1 (3, 4), carbapenems appear to be becoming the last line of defense in the treatment of multidrug-resistant bacteria (5).

To combat the serious challenge posed by MDR, new antibacterial approaches are being sought. These approaches include (i) the synthesis and design of new antibacterial materials (6–10), (ii) the discovery of new uses for old drugs (11–14), (iii) the construction of new drug delivery vehicles (9, 15, 16), and (iv) vaccine development (17–19). AMPs have attracted considerable attention as antimicrobials. The main sources of AMPs are bacteria, archaea, animals (20), fungi, and plants (21). However, the relationship between secretion systems and AMPs remains unclear. In this study, we identified a type VI secretion system (T6SS) effector in extraintestinal pathogenic Escherichia coli (*ExPEC*) RS218 and modified it to create an AMP. The results showed that the modified AMPs have a significant inhibitory effect on the growth of bacteria.

T6SS is a protein-delivering nanoweapon that has been found to be present in approximately one-quarter of fully sequenced Gram-negative bacteria (22). Typically, T6SS is involved in the competition between eukaryotes and prokaryotes for survival. When bacteria encounter adversity, they sense the adverse stimuli and rapidly proceed to secrete effectors.

Typically, T6SS secretes effectors that have bactericidal action (23). Some effectors of the T6SS have specific structures; one typical effector structure is a proline-alanine-alanine-arginine (PAAR) structural domain at the N terminus. Effectors with N-terminal PAAR characteristics have variable C-terminal structures (24), and this variability causes the effectors to have different functions. In this paper, we identified an effector in *ExPEC* RS218 with an N-terminal PAAR structure characterized by bacteriocins at its C terminus. Based on this effector, we designed and validated three AMPs that exhibit antimicrobial activity, particularly against Gram-positive bacteria.

Type VI secretion system-related antibacterial peptide (Tsap) AMPs exert a bactericidal effect mainly by disrupting cell membranes and increasing reactive oxygen species (ROS) levels within bacteria. Tsap binds more strongly to lipoteichoic acid (LTA) of Gram-positive bacteria than to lipopolysaccharide (LPS) of Gram-negative bacteria and therefore has a stronger inhibitory effect on Gram-positive bacteria. This study broadens the identified sources of AMPs and describes the modification of AMPs for use as T6SS effectors.

## RESULTS

### Tsap is a T6SS effector in *ExPEC* RS218.

**FIG 1:**
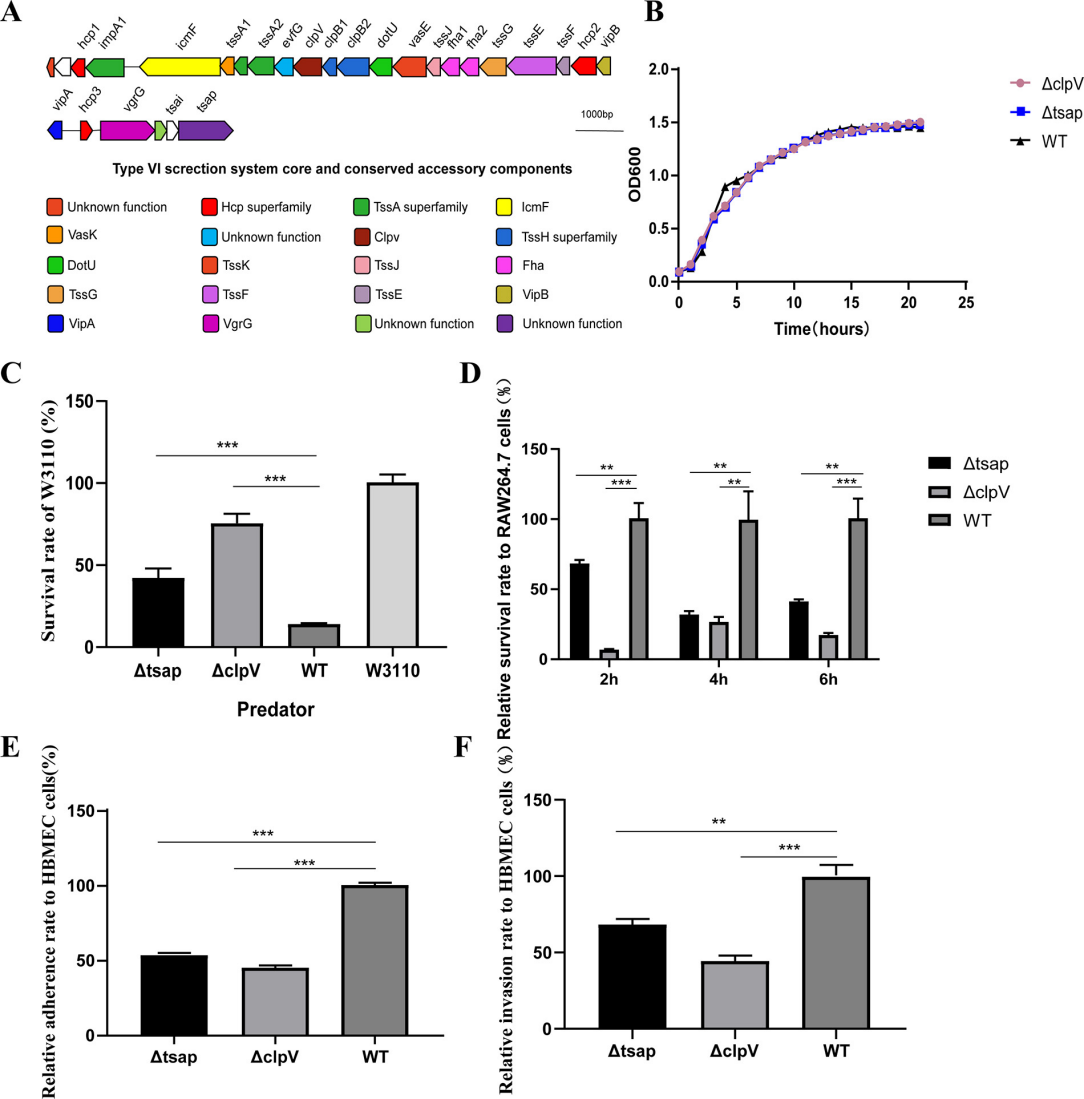
Tsap is a T6SS effector in *ExPEC* RS218. (A) Schematic diagram of the T6SS gene of RS218. Tsap is located at the end of the T6SS cluster. (B) Growth curves of the Δtsap, ΔclpV, and WT strains. The curves were obtained using a fully automated growth curve analyzer (CFP-1100-C; Finland). (C) Competitive ability of the Δtsap strain. The mutant and WT strains were used as predators, and E. coli W3110 was used as prey. The predator and prey were cocultured at a ratio of 1:10 at 30°C for 12 h; 10× serial dilutions were then prepared, and CFUs were counted. (D) Determination of antimacrophage capacity. RAW 264.7 cells were infected with mutant or WT strains at an MOI of 10. After 2 h, 4 h, or 6 h, intracellular bacteria were counted in 10× serial dilutions of the cultures. Adhesion (E) and invasion (F) capabilities of Δtsap are shown. All experiments were repeated three times independently. **, *P* < 0.01; ***, *P* < 0.001.

In *ExPEC* RS218, we found a classical T6SS cluster that has a gene with unknown function (gene 1835) located downstream of it (Fig. 1A). The presence of a typical PAAR domain in the 1835 N terminus suggests that this gene might be a potential T6SS effector. We knocked out 1835 and the T6SS essential assembly gene clpV and measured the growth curves (Fig. 1B) of Δ1835, ΔclpV, and the wild-type (WT) strain. There were no differences in the growth curves of the three strains. The results of bacterial competition assays designed to evaluate T6SS function indicated that the survival rate of the prey strain W3110 in the Tsap mutant strain group was significantly increased compared with that of the WT competition group. A similar tendency was observed for the ΔclpV competition group (Fig. 1C). Antiphagocytic activity (Fig. 1D), adhesion, and invasion ability were correspondingly decreased (Fig. 1E and F). These results further suggested that 1835 is a T6SS effector. We recommend that this gene be renamed “type VI secretion system-related antibacterial peptide” (Tsap).

**FIG 2:**
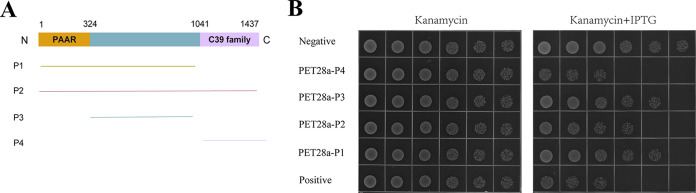
The C terminus of 1835 has antibacterial activity. (A) Schematic diagram of the 1835 protein. The PAAR region of the peptide is shown in yellow, the middle fragment is shown in blue, and the C39 peptide region is shown in purple. (B) Growth of 10× serial dilutions of bacteria on Luria Bertani agar (LBA) plates and on LBA plates containing 50 μg/mL kanamycin and 0.5 mM isopropyl-β-d-thiogalactopyranoside (IPTG). The negative control is BL21(DE3) containing an empty PET28a plasmid. The positive control is Rhs-3CT in *ExPEC* PCN033.

According to the NCBI BLASTP website, the C terminus of 1835 is speculated to be a C39 peptidase. We divided the sequence of 1835 into three parts (Fig. 2A), as follows: a PAAR domain (1 to 324 bp), a C39 peptidase family domain (1041 to 1437 bp), and a middle portion (324 to 1041 bp). Then by detecting cell expression toxicity, according to Fig. 2B, we found that cells show obvious growth inhibition only if the sequences are in the C terminus (Fig. 2B). The C-terminal overexpression strain showed obvious growth inhibition, indicating that this portion of the gene might encode a peptide that is cytotoxic to prokaryotes. We also noted that this portion of the peptide has an α-helical structure similar to that found in most AMPs. Based on these findings, we wondered whether this part of the sequence could be modified to produce an AMP.

### Antimicrobial activity.

We designed nine peptides and measured the MICs of those peptides. According to Luna et al. (25), in a nutrient-limited situation, antibacterial peptides confer significantly enhanced antibacterial activity. We measured the antibacterial activity of peptides in RPMI 1640 medium and in RPMI 1640 medium supplemented with amino acids; the MIC results are presented in Table 1. We chose the three peptides that exhibited significant antibacterial activity for further research. These peptides have excellent antibacterial and bactericidal effects against both Gram-negative and Gram-positive bacteria. In comparison to MICs in a single RPMI 1640 medium or other amino acid supplement solution, the inhibitory activity of the bacteria increased with the presence of 0.5 mM l-arginine, 0.5 mM glycine, or nonessential amino acid solutions. Both Tsap1 and Tsap3 significantly inhibited the growth of standard strains, particularly in Staphylococcus aureus ATCC 25923. Tsap1 and Tsap3 showed a superior MBC effector, which is 4 to 8 g/mL, compared with Tsap2, which had an MBC of 16 to 32 g/mL. Tsap AMPs also show significant bactericidal activity against MDR strains. The results are presented in Table 2, and the AMPs all have a broad spectrum of antimicrobial activity.

**TABLE 1:** AMP antimicrobial activity against laboratory strains

Parameter	Antimicrobial activity^*a*^ (μg/mL)					
	E. coli ATCC 25922			S. aureus ATCC 25923		
	Tsap1	Tsap2	Tsap3	Tsap1	Tsap2	Tsap3
RPMI	32	128	32	16	64	16
Amino acid supplementation in RPMI						
Nonessential amino acid solution	16	64	16	16	64	16
l-Arginine (0.5 mM)	16	64	16	16	16	16
Glycine (0.5 mM)	16	64	16	16	64	16
Leucine (1 mM)	32	128	32	16	64	16
l-Histidine (0.5 mM)	32	128	32	16	64	16
l-Tryptophan (0.125 mM)	32	128	32	16	64	16
MBC	4	32	4	4	16	8

a All values are MIC unless otherwise specified.

**TABLE 2:** AMP antimicrobial activity against MDR bacteria

Bacterial strain	Antimicrobial activity (μg/mL) of:					
	Tsap1		Tsap2		Tsap3	
	MIC	MBC	MIC	MBC	MIC	MBC
S. aureus USA300	16	2	16	8	16	2
S. aureus 1802043	16	2	16	8	16	2
S. aureus USA200	64	8	512	64	64	16
S. aureus ATCC 43300	16	4	16	8	16	4
Streptococcus SC19	16	4	16	8	16	4
Bacillus subtilis NCD-2	16	4	16	8	16	4
*ExPEC* RS218	64	32	256	64	64	32
*ExPEC* PCN033	64	32	256	64	64	32

### Antimicrobial peptide design and characteristics.

**FIG 3:**
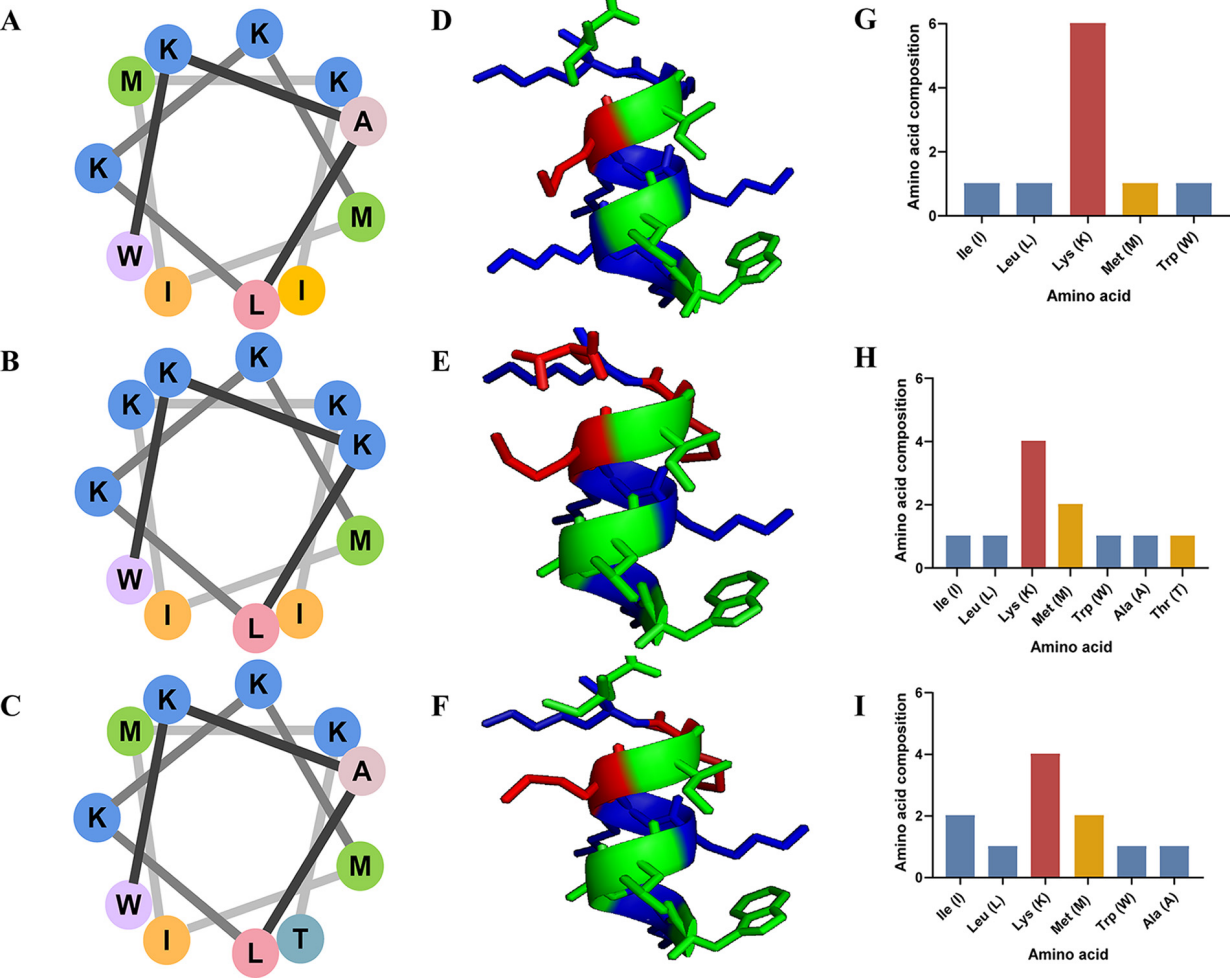
Antimicrobial peptide and characteristics. Spiral wheels for Tsap-1 (A), Tsap-2 (B), and Tsap-3 (C). Different colors indicate different types of amino acids; hydrophobic amino acids are shown in peach, yellow, green, pink, and purple; alkaline amino acids are shown in blue; and neutral (uncharged) amino acids are shown in bluish green. The 3D model of Tsap-1 (D), Tsap-2 (E), and Tsap-3 (F). The amino acid assembly of Tsap-1 (G), Tsap-2 (H), and Tsap-3 (I) is shown. Alkaline amino acids were shown in red, hydrophobic uncharged residues were colored in yellow, and polar uncharged amino acids were marked in blue.

To provide a better understanding of the nature of the AMPs, we have listed the sequence, number of amino acids, molecular weight, pI, and hydrophobicity (negative values indicate hydrophilicity, and smaller values indicate greater hydrophilicity) in Table 3. In the spiral wheel plots for Tsap-1 (Fig. 3A), Tsap-2 (Fig. 3B), and Tsap-3 (Fig. 3C), different amino acids are shown in different colors. The amino acid linkage sequence is indicated by a progression from black to gray. Through model analysis, we found that the hydrophobic amino acids, which are shown in green, lie in the same plane and that the neutral and positively charged amino acids (shown in blue and red, respectively) lie in another plane (Fig. 3D, E, and F). The numbers of different amino acids are shown in Fig. 3G, H, and I.

**TABLE 3:** Basic properties of AMPs

Name	Sequence	No. of amino acids	mol wt	Theoretical pI	Net charge	GRAVY^*a*^
Tsap1	WKKLKKMIKK	10	1,330.78	10.70	6.0	−1.41
Tsap2	WKALKKMIMKT	11	1,377.81	11.01	4.0	−0.3
Tsap3	WKALKKMIMKI	11	1,389.87	11.01	4.0	0.17

a GRAVY indicates grand average of hydropathy.

### Hemolytic activity and cytotoxicity.

**FIG 4:**
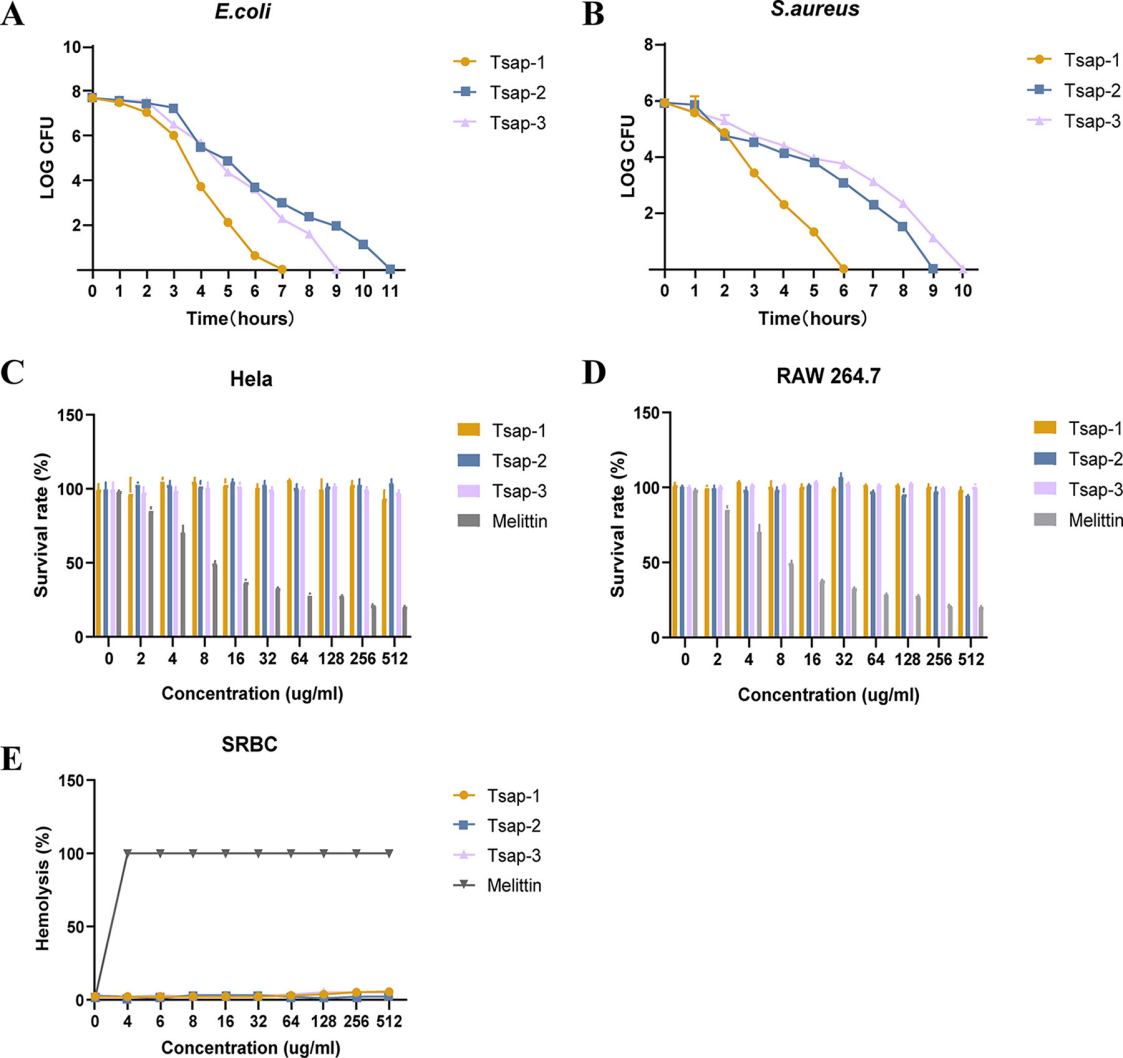
Hemolytic activity and cytotoxicity. Shown are time-kill curves for Tsap AMPs (1× MBC) in E. coli ATCC 25922 (A) and S. aureus ATCC 25923 (B). Cytotoxicity of AMPs at various concentrations in HeLa cells (C) and RAW 264.7 cells (D) is shown. Notably, 0 μg/mL to 512 μg/mL of melittin were used as a positive control, and the PBS group was used as a negative control. Hemolytic activity toward sheep red blood cells of Tsap peptides at various concentrations are shown (E), and melittin was used as the control.

In our measurements of the MICs of specific peptides against standard and clinical bacterial strains, we found that Tsap peptides have good efficiency in inhibiting or killing bacteria, especially Gram-positive bacteria. The time-kill curves showed that Tsap-1 can kill E. coli and S. aureus, especially S. aureus, within 7 h. Tsap-2 and Tsap-3 also showed significant bactericidal activity (Fig. 4A and B).

We also measured the cytotoxicity of the peptides to HeLa cells and mouse RAW 264.7 cells, compared with a positive-control melittin-treated group, and the peptides showed only negligible toxicity (Fig. 4C and D). Sheep red blood cells were used to measure the hemolytic activity of those peptides. The results showed that there was only slight hemolytic activity at high peptide concentrations (Fig. 4E). But the group treated with 4 μg/mL melittin already showed complete hemolysis activity.

### Tsap peptides disrupt the membranes of E. coli and S. aureus.

**FIG 5:**
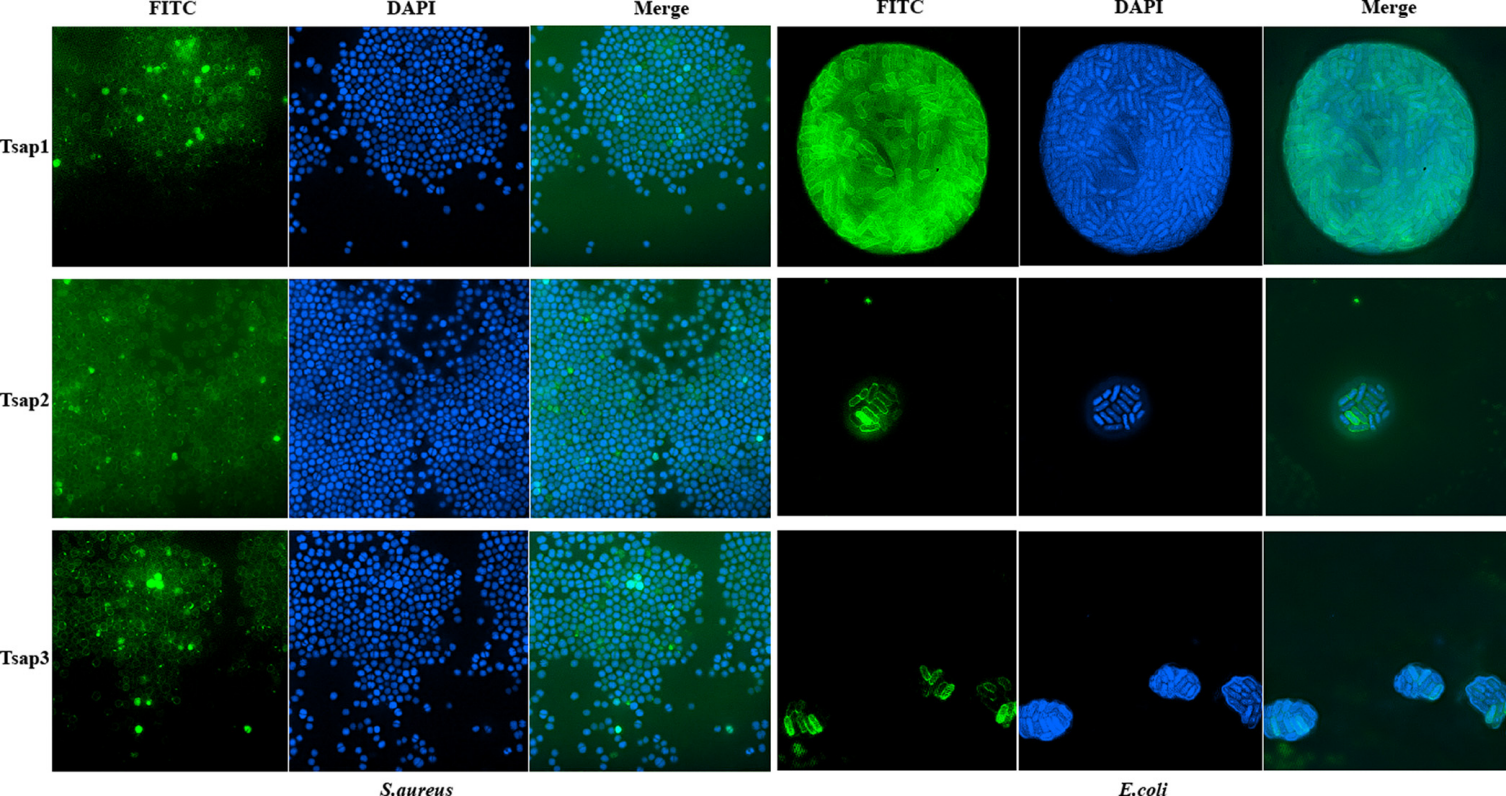
Colocalization of FITC-labeled Tsap AMPs with E. coli and S. aureus. Bacteria at mid-log phase were incubated with 0.5× MBC FITC-labeled AMPs for 30 min at 37°C. The bacteria were then incubated with DAPI for 15 min and observed using SIM, and images were acquired.

To determine the mechanism through which the peptides exert their antibacterial activity, we tagged the peptides with fluorescein isothiocyanate (FITC). Using structured illumination microscopy (SIM), which has a higher resolution than confocal or fluorescence microscopy and can visualize the structure of bacteria, we found that the peptides are first located in bacterial membranes and then enter the bacteria (Fig. 5). About all the bacterial membrane was covered by FITC-tagged AMPs after being treated with 0.5 MBC AMPs for 30 min. This finding suggests that Tsap peptides could damage the cell membrane since certain AMPs even reach the membrane of bacteria.

**FIG 6:**
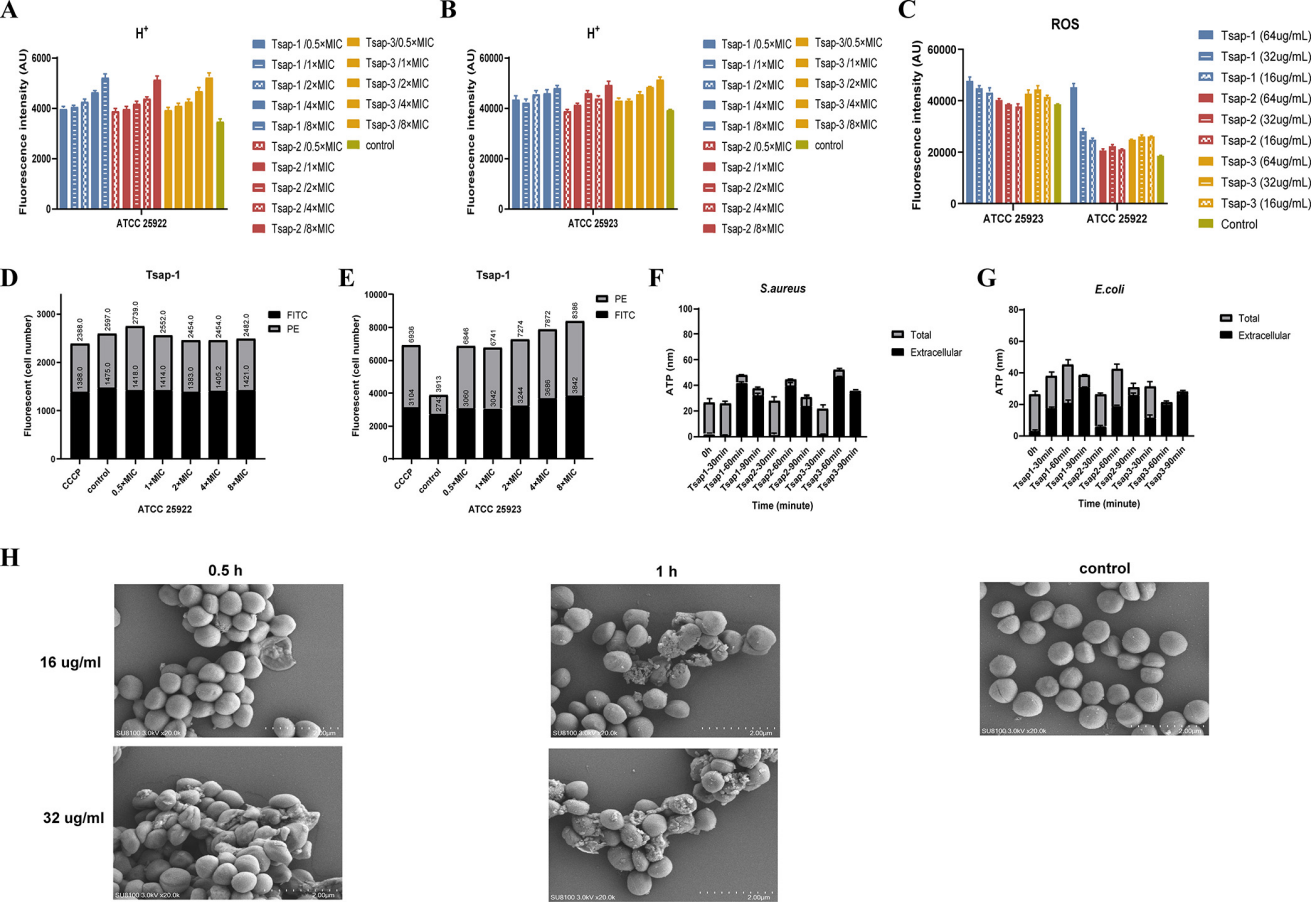
Tsap peptides disrupt the membranes of E. coli and S. aureus. The H+ content of ATCC 25922 (A) and ATCC 25923 (B) after treatment with Tsap peptides at various concentrations is shown. Untreated bacteria were used as a control. (C) ROS levels in ATCC 25922 and ATCC 25923 after treatment with Tsap AMPs at various concentrations. The effect of Tsap on membrane permeability in Escherichia coli (D) and Staphylococcus aureus (E) is shown. FITC staining and flow cytometry were used to determine the number of bacteria with altered membrane potential (PE) and the number of unaltered bacteria after exposure to Tsap-1 at 0.5× MIC, 1× MIC, 2× MIC, 4× MIC, and 8× MIC. The CCCP-treated group was used as a positive control. Determination of ATP leakage in S. aureus (F) and E. coli (G) treated with AMPs for different amounts of time is shown. The bacterial cells were treated with Tsap AMPs at 1× MIC for 30 min, 60 min, and 90 min, and intracellular and extracellular ATP contents were then determined. (H) Shows scanning microscopy images of Tsap-1-treated S. aureus. Scanning microscopy images of S. aureus ATCC 25923 are also shown after treatment with Tsap-1 at 16 μg/mL or 32 μg/mL for 0.5 h or 1 h, respectively. Untreated ATCC 25923 were used as a negative control.

We next used the 2',7'-bis-(2-carboxyethyl)-5-(and-6)-carboxyfluorescein, acetoxymethyl ester (BCECF-AM) probe to measure the hydrogen ion (H^+^) content of E. coli and S. aureus cells. Bacteria use respiratory enzymes that are membrane bound to power their energy metabolism. These enzymes absorb chemical energy and convert it across their cell membranes using H^+^ or Na^+^ proton pumps. The integrity of the bacterial membrane is thus represented by the H^+^ content (26). As the concentration of the peptide was increased, the H^+^ content increased in the Tsap peptide-treated group (Fig. 6A and B), which indicates that Tsap can break the membrane integrity. Several publications claim that various AMPs can cause cell death by raising ROS levels (27) and that ROS levels are also related to the structural integrity of cell membranes (28). The peptides promoted an increase in ROS content (Fig. 6C) and thereby caused bacterial death. These phenomena were especially obvious in S. aureus.

The 3,3'-diethyloxacarbocyanine iodide [DIOC_2_(3)] fluorescent probe produces green fluorescence in bacteria under normal conditions. Higher membrane potentials lead to an aggregation of the dye, causing an increase in red fluorescence. If the membrane potential is altered by disruption of proton channels, less red fluorescence is observed. The flow cytometry results showed that the amount of red fluorescence decreased in E. coli ATCC 25922 cells exposed to higher Tsap-1 concentrations. Similar results were obtained in the positive-control carbonyl cyanide *m*-chlorophenylhydrazone (CCCP)-treated group, indicating that Tsap-1 targets the proton motive force in E. coli. However, in S. aureus, the amount of red fluorescence increased in both the CCCP-treated group and the Tsap-1-treated group, indicating that a change in membrane potential had occurred (Fig. 6D and E).

Next, we performed an ATP leakage assay in which we measured the total and extracellular ATP content of bacteria that had been treated with Tsap peptides for various amounts of time. In S. aureus and E. coli, the extracellular ATP content increased over time (Fig. 6F and G).

Scanning microscopy was used to visualize membrane integrity in S. aureus (Fig. 6H). In the negative-control group, the membranes of S. aureus were intact and smooth. However, with exposure to increasing concentrations of peptide for longer times, the bacterial film crumpled or broke.

The experiments described above show that Tsap peptides are able to disrupt the integrity of cell membranes, especially in S. aureus.

### Tsap peptides bind preferentially to S. aureus lipoteichoic acid (LTA).

**FIG 7:**
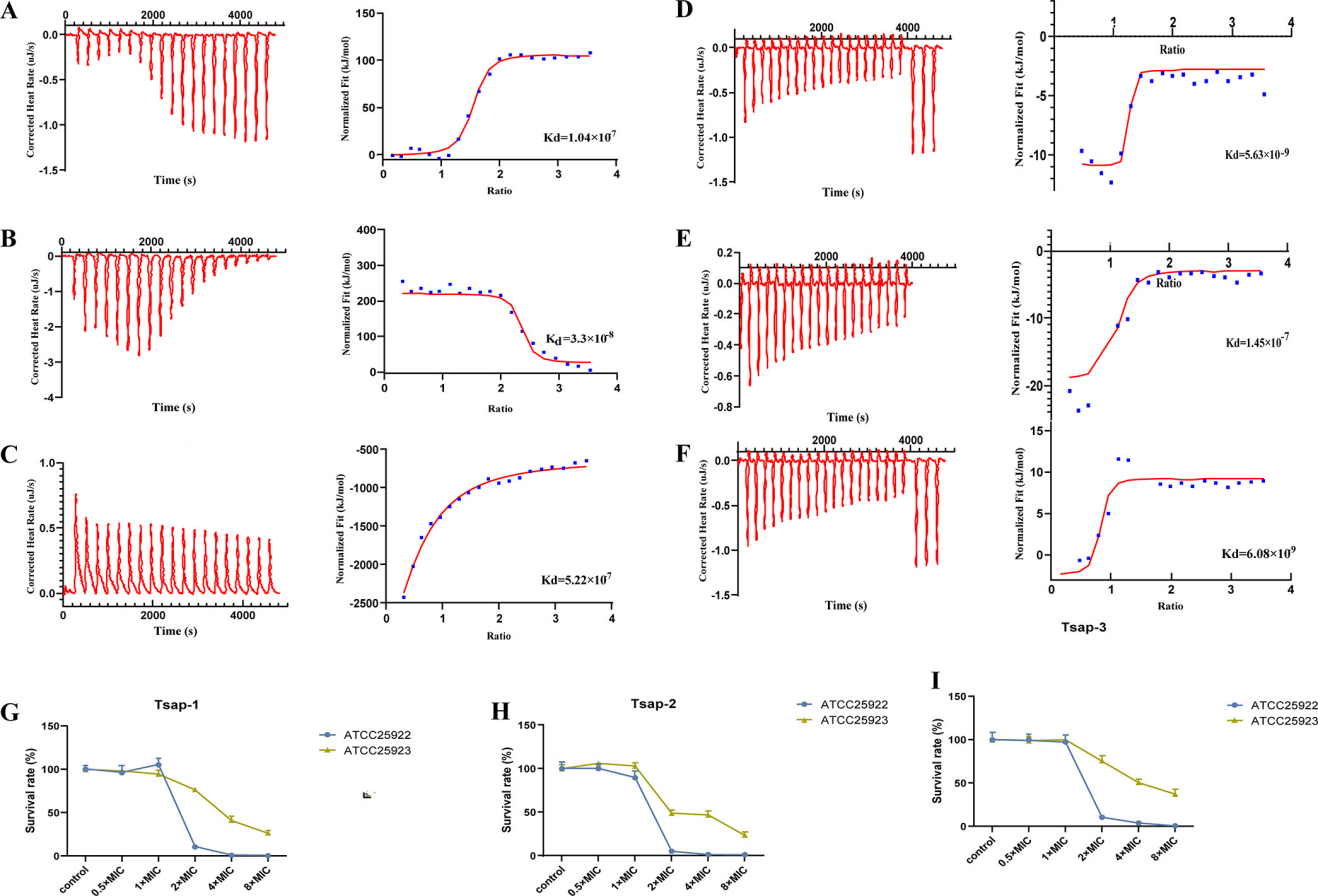
Tsap peptides exhibit preferential binding to S. aureus LTA. The ITC results of LPS binding to Tsap-1 (A), Tsap-2 (B) and Tsap-3 (C). ITC results for Tsap-1 (D), Tsap-2 (E), and Tsap-3 (F) and LTA are shown. The original titration data and the integrated heat measurements are shown in the left and right plots, respectively. Competitive bactericidal activity of Tsap-1 (G), Tsap-2 (H), and Tsap-3 (I) between LPS and LTA. S. aureus ATCC 25923 cells were grown to mid-log phase, washed three times with PBS and adjusted to an OD_600_ of 1.0. LPS (1 mg/mL) and LTA (1 mg/mL) were added separately, and various concentrations of AMPs were added. After incubation at 37°C for 2 h, 10× serial dilutions were prepared, and CFU were counted.

LTA is a membrane component of Gram-positive bacteria, and its role is comparable to that of LPS in Gram-negative bacteria. We used isothermal titration calorimetry (ITC) to detect the bonding energy of peptides to the E. coli and S. aureus membrane components LPS (Fig. 7A to C) and LTA (Fig. 7D to F). The binding energy of the Tsap peptides to LTA was much higher than that of their binding to LPS at the same concentrations.

We then performed a competitive bactericidal assay in which we added 1 mg/mL LPS or 1 mg/mL LTA to cultures of ATCC 25923. As the concentration of Tsap peptides was increased, the survival of the LPS-neutralized groups obviously decreased (Fig. 7G to I). However, there was no obvious bactericidal effect in the LTA-neutralized groups. This result means that LTA neutralized most of the peptides and indicates that Tsap peptides have a higher affinity for LTA than for LPS.

### Tsap peptides suppress LTA-stimulated inflammation *in vitro* and *in vivo*.

**FIG 8:**
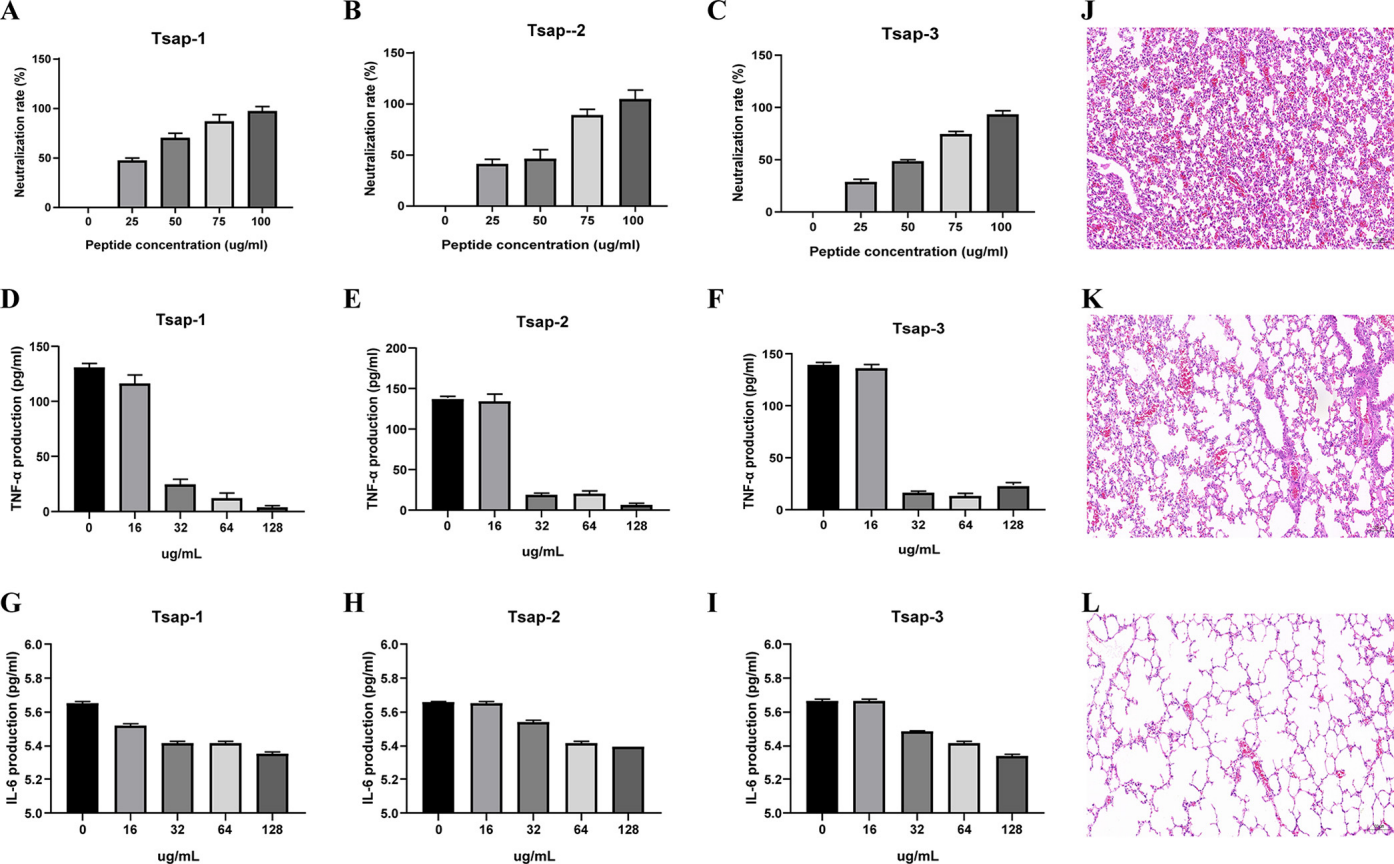
Tsap peptides suppress LTA-stimulated inflammation *in vitro* and *in vivo*. The ability of Tsap-1 (A), Tsap-2 (B), and Tsap-3 (C) to neutralize endotoxins at various concentrations is illustrated. TNF-α content in the Tsap-1-treated (D), Tsap-2-treated (E), and Tsap-3-treated (F), LTA-infected inflammation model. IL-6 content in Tsap-1-treated (G), Tsap-2-treated (H), and Tsap-3-treated (I), LTA-infected HeLa cell inflammation models. LTA-mediated lung inflammation in mice (J) and Tsap-1-treated sections (K); the untreated group was used as a control (L).

Both LPS and LTA induce inflammation similar to the inflammation that occurs during the bacterial infection process (29). Our results showed that the endotoxin was neutralized as the Tsap concentration increased, and at concentrations approaching 100 μg/mL, endotoxin was almost completely neutralized (Fig. 8A to C) (30, 31). After treatment of HeLa cells with 200 ng/mL LTA for 8 h, the levels of tumor necrosis factor alpha (TNF-α) (Fig. 8D to F) and interleukin-6 (IL-6) (Fig. 8G-I) in the Tsap peptide-treated group were much lower than those in the untreated group. This effect was enhanced as the concentration of Tsap was increased. After measuring the levels of TNF-α and IL-6 *in vitro*, we measured the effects of peptides *in vivo*.

We used the LTA-induced mouse endotoxemia model to detect inflammation in lung tissue (Fig. 8J to L). LTA pulmonary inflammatory mice displayed significant alveolar epithelial and capillary endothelial cell injury, alveolar edema, and inflammatory cell infiltration. The treated group showed a significant reduction in those changes. The results indicate that Tsap AMPs can alleviate LTA-stimulated inflammation *in vitro* and *in vivo*.

### Tsap-1 has a good combined bactericidal effect *in vitro* and *in vivo*.

Ciprofloxacin is a broad-spectrum antibacterial drug that is active against Gram-negative and Gram-positive bacteria and is used widely in the treatment of bacterial infections (31). We found previously that the MICs of ciprofloxacin, Tsap-1, Tsap-2, and Tsap-3 in S. aureus USA200 were 16 μg/mL, 64 μg/mL, 512 μg/mL, and 64 μg/mL, respectively. The effects of the administration of Tsap peptides in combination with ciprofloxacin are shown as a checkerboard plot. The fractional inhibitory concentration indices (FICIs) measured in the assays were 0.5, 1.125, and 1.25. Only Tsap-1 showed a synergistic effect with ciprofloxacin; no differences were observed when Tsap-2 or Tsap-3 was combined with ciprofloxacin.

**FIG 9:**
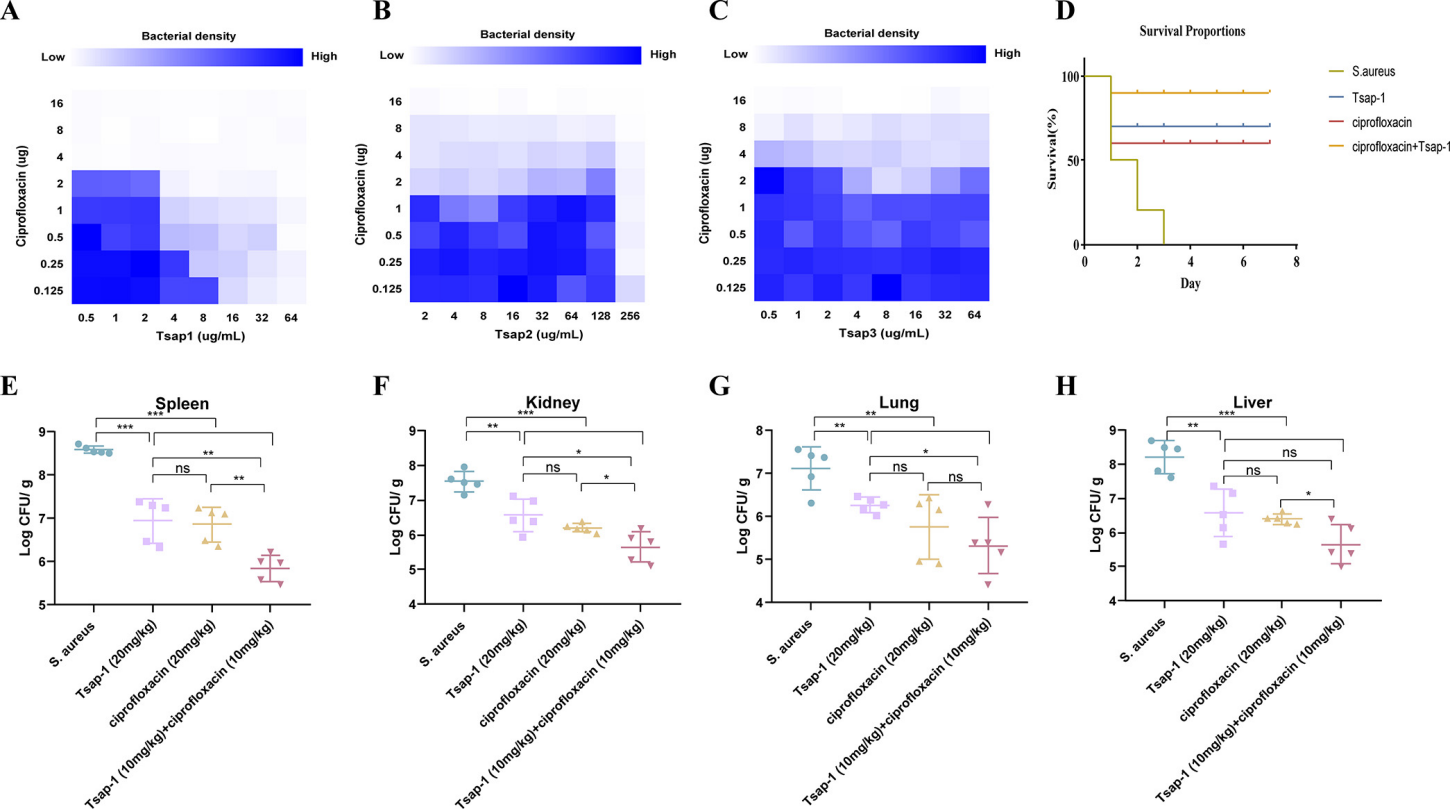
Tsap-1 exhibits good combined bactericidal effects *in vitro* and *in vivo*. Schematic checkerboard diagram showing the results obtained when Tsap-1 (A), Tsap-2 (B), and Tsap-3 (C) were administered in combination with ciprofloxacin. The blue color ranging from light to dark indicates an increase in drug concentration. Survival of mice after S. aureus USA200 injection in the ciprofloxacin, Tsap-1, and combination treatment groups (D). Bacterial load in spleen (E), kidney (F), lung (G), and liver (H) in mice infected with S. aureus USA200 after treatment with ciprofloxacin, Tsap-1, or a combination of the two.

In the S. aureus-infected mouse model, all of the mice in the untreated group died within 3 days. Three mice in the Tsap-1-treated group and four mice in the ciprofloxacin-treated group died within 7 days, indicating 70% and 60% protection, respectively, by the drug. However, in the group that was treated with a combination of Tsap-1 and ciprofloxacin, 90% protection was achieved (Fig. 9B).

After infection with S. aureus USA200, mouse tissue CFU loads were counted. Although the CFU load of the Tsap-1-treated group did not differ from that of the ciprofloxacin treatment group, the bacterial loads in all tissues of the animals in the treated groups reduced by about 32 times compared with those of the untreated group, and those of the animals treated with a combination of the two drugs decreased by 1,000-fold. The experiments described above show that Tsap-1 has good *in vivo* and *in vitro* bactericidal effects and that it has a strong antibacterial effect when administered in combination with ciprofloxacin.

## DISCUSSION

AMPs are naturally abundant peptides that can be obtained from a wide range of sources and vary in length from a few to several tens of amino acid residues. T6SS is a nanoweapon that is present in many Gram-negative bacteria, and one of its functions is to secrete effectors that have bactericidal properties. Normally, bacteria evolve corresponding proteins to protect themselves. Although the direct expression of such proteins is of little significance, it indicates that they have the potential to be modified into AMPs. In this study, we identified a T6SS effector (Tsap) in *ExPEC* RS218, modified it after sequence analysis, and investigated the antimicrobial mechanisms of action of the three resulting AMPs (Tsap-1, Tsap-2, and Tsap-3).

In addition to their structural differences, AMPs also differ in their physicochemical properties (32). The α-helix is one of the most typical structures in AMPs, and the original amino acid sequence of Tsap “WDALKKMIMDT” was modified into three AMPs. Sequence analysis and the construction of models of the three AMPs showed that all three AMPs have α-helical structures and consist mainly of hydrophobic and neutral amino acid residues (33). Both Tsap-1 and Tsap-2 are hydrophilic, and Tsap-1 is especially hydrophilic. In general, cationic AMPs have better antimicrobial activity than noncationic AMPs. All three of the modified AMPs are positively charged, which is one of the reasons for the good antimicrobial efficacy of these three antimicrobial peptides.

Furthermore, we investigated the antibacterial mechanism of action of the Tsap AMPs. We found that all three AMPs have good antibacterial and bactericidal activity against both Gram-positive bacteria and Gram-negative bacteria. The effect on Gram-positive bacteria was particularly pronounced. It was manifested mainly by membrane damage and an elevation of ROS levels. The AMPs had a stronger scavenging effect on Gram-positive bacteria due to their higher affinity for LTA.

The LPS of Gram-negative bacteria plays an important role in the inflammatory process, and the LTA component of Gram-positive bacteria has a role similar to that of LPS (34). We first assessed the toxicity of Tsap AMPs in cytotoxicity assays. The three peptides were found to have almost no toxic effects on cells. Second, through the establishment of an *in vitro* LTA cell inflammation model, we found that Tsap alleviates the inflammation caused by LTA. In addition, the three peptides alleviated lung inflammation caused by the administration of LTA to mice.

The kidneys' ability to metabolize substances can be diminished by bacterial infections and chronic diseases; in individual with these conditions, antibiotic doses must be reduced, and bacterial killing is achieved by other means (35). To assess whether the Tsap AMPs were effective in combination with first-line drugs, we used ciprofloxacin in combination with the AMPs against the MDR strain S. aureus USA200. Only Tsap-1 displayed synergy with ciprofloxacin. The alleviating effect of the AMPs on lung infections caused by LTA suggests that these three peptides may have *in vivo* antimicrobial activity. We infected mice with S. aureus USA200 and found a significant decrease in the tissue load of S. aureus in mice in the Tsap-1 and ciprofloxacin treatment groups, but there was no significant difference between these two groups. This finding suggests that both AMP and ciprofloxacin have a good *in vivo* clearance effect. The animals that received a combined treatment with Tsap-1 and ciprofloxacin showed better bacterial clearance, indicating that the AMP and the antibiotic had a synergistic antibacterial effect.

In summary, this study identified and modified the T6SS effector into the following three positively charged alpha-helical peptides: Tsap-1, Tsap-2, and Tsap-3. These peptides have good antibacterial and bactericidal effects on Gram-positive bacteria and Gram-negative bacteria. The first step in the antibacterial mechanism of Tsap AMP is molting on the cell membrane and interaction with LPS or LTA. This process leads to ion efflux and bacterial death through alterations in membrane potential and ion permeability. Bacterial death is then promoted by the elevation of intracellular ROS levels. During this period, LPS and LTA released by bacteria can be neutralized by Tsap, yielding an anti-inflammatory effect. The synergistic effect of this AMP with antibiotics also suggests that Tsap can function as an antimicrobial additive.

## MATERIALS AND METHODS

### Bacteria and materials.

The strains used in this work are listed in Table S1 in the supplemental material. E. coli, Bacillus subtilis NCD-2, and S. aureus were cultured in LB broth, and Streptococcus was cultured in Trypticase soy broth (TSB) supplemented with 10% fetal bovine serum (FBS) at 37°C. RPMI 1640 medium was purchased from Gibco (USA). Chloromycin, isopropyl-β-d-thiogalactoside, 2′,7′-dichlorofluorescein diacetate (H_2_DCFDA), ciprofloxacin, LPS, and LTA were purchased from Merck (USA).

### Gene manipulation.

CRISPR-Cas9-mediated mutant construction was performed as described previously (36). *ExPEC* RS218 was transformed with pCas, and an LBA plate containing 50 μg/mL kanamycin was used to select the positive strain. Electrocompetent receptor cells were prepared in 10 mM l-arabinose. We used the RS218 genome as a template and primer up-1 (GGGGTTGGGCCAGACGGTGAATGTG) and primer up-2 (ACCATCCCCGATATGATAGTTGCTTATTGATTCCTGAATA) to obtain the upstream homologous arm and primer down-1 (TATTCAGGAATCAATAAGCAACTATCATATCGGGGATGGT) and primer down-2 (CATTTTTAACCTCAGGTGAAATA) to obtain the downstream homologous arm. Fusion PCR was used to connect the upstream and downstream homologous arms. We then used the pTarget plasmid as a template, and primer-1 (GGTAGTGCTCATGCCCCTTCGTTTTAGAGCTAGAAATAGC) and primer-2 (GAAGGGGCATGAGCACTACCACTAGTATTATACCTAGGAC) were used to obtain the recombinant plasmid. The fusion homologous arm and the recombinant plasmid were then transformed into the pCas-positive strain. After culture at 30°C overnight in 50 μg/mL kanamycin and 50 μg/mL spectinomycin, the recombinant strain was identified by PCR.

### Bacterial competition assay.

Bacterial competition assays were performed as described previously (37). The mutant and WT strains were used as predators, and E. coli W3110 was used as prey. Plasmid PHSG396 containing a chloramphenicol resistance gene was transferred into W3110. The bacteria were then incubated until the culture reached an optical density at 600 nm (OD_600_) of 0.9, washed three times with phosphate-buffered saline (PBS), adjusted to an OD6_00_ of 0.5, and mixed at a predator:prey ratio of 1:10. The mixture was spotted onto a 50% LBA nitrocellulose membrane (Millipore). After incubation on plates at 30°C for 12 h, the bacteria were washed and counted after 10-fold serial dilution. The experiment was repeated at least three times.

### Measurement of adhesion, invasion, and antiphagocytic activity.

Adhesion and invasion were assessed as described previously (38). Human brain microvascular endothelial cells (HBMECs) were inoculated into 24-well cell culture plates. After the cells had covered the bottom of the plate, they were cultured in RPMI 1640 medium without FBS for 30 min. The bacterial concentration was then adjusted, and the cells and bacteria were cocultured at a multiplicity of infection (MOI) of 10. For the invasion assay, the plates containing the cells were centrifuged at 1,000 × *g* for 10 min (this step was omitted in the adhesion assay). The mixture was then incubated for 1 h. For the invasion assay, 100 μg/mL gentamicin was added, and the cells were incubated for 1 h. The plates were then washed three times with PBS and reincubated in 1640 medium supplemented with 5% FBS for 1 h. After three washes with PBS, the cells were lysed with 0.025% Triton X-100, and 10-fold dilutions were plated onto smear plates and counted. The experiment was repeated at least three times.

Antiphagocytic activity was assessed as described previously with some modifications (39). In brief, mouse macrophage RAW264.7 cells were cultured and plated onto 24-well plates. When the cells had covered the bottom of the plate, the cultures were incubated in Dulbecco’s modified Eagle’s medium (DMEM) without FBS for 30 min. The bacterial concentration was then adjusted, and the cells were cocultured with bacteria at an MOI of 10. The plates containing the cells were then centrifuged at 1,000 × *g* for 10 min. After incubation for 1 h, the cells were washed three times with PBS and incubated in DMEM containing 100 μg/mL gentamicin for 1 h, followed by three washes with PBS. The cells were then incubated for 2, 4, or 6 h; washed three times with PBS; lysed in 0.025% Triton X-100; and diluted and spread onto plates for counting. Survival was calculated using the following equation: [(CFU/mL) *t* = 2/4/6 h/(CFU/mL) *t* = 0 h] × 100%.

### Peptides.

Tsap-1 (WKKLKKMIKKWKKLKKMIKK), Tsap-2 (WKALKKMIMKT), Tsap-3 (WKALKKMIMKIWKALKKMIMKI), and melittin (GIGAVLKVLTTGLPALISWIKRKRQQ) were synthesized by the GenScript Biotechnology Company (Nanjing, China).

### MIC and MBC.

MIC was measured according to Luna et al. (25) as described previously. Briefly, 100 μL of RPMI 1640 medium containing drugs at various concentrations was cultured with 100 μL of bacteria at 1 × 10^6^ CFU/mL in 96-well plates at 37°C for 18 to 20 h. Wells containing 100 μL of bacteria at 1 × 10^6^ CFU/mL and 100 μL medium were used as positive controls. Wells containing 100 μL drug and 100 μL medium were used as drug-negative controls, and wells containing 200 μL medium were used as negative controls.

In amino acid-supplemented RPMI 1640 medium, 100× nonessential amino acid solution, l-arginine (0.5 mM), glycine (0.5 mM), leucine (1 mM), l-histidine (0.5 mM), and l-tryptophan (0.125 mM) were added separately.

The MBC, in which a combination of 10 μL from MIC was put into Mueller-Hinton agar medium and incubated for 24 h, was the first one in which bacterial growth was seen.

### Time-kill curve assay.

The time-kill curve assay was performed as described previously (40). Briefly, bacteria that had been cultured overnight were transferred to LB broth, grown to an OD_600_ of 0.5, washed three times with PBS, and adjusted to 1 × 10^6^ cells/mL. Peptide (1× MBC) was added to the bacterial culture, and 100-μL aliquots of the culture were removed hourly thereafter. The CFUs were counted after 10× serial dilution.

### Cytotoxicity and hemolytic activity.

The cytotoxicity of the peptides to HeLa and RAW 264.7 cells was measured using CCK8. Cells were seeded in 96-well plates at 10^4^ cells/well, and various concentrations of Tsap peptides were added to the fully grown cells. Cells that had been exposed to melittin at various concentrations were used as positive controls, and wells containing PBS only were used as negative controls. After incubation for 2 h, the cells were washed three times with PBS, and the medium was replaced with fresh medium containing 10% CCK8 solution. After incubation of the cells for an additional 3 h, the OD_590_ of the culture was measured using a microplate reader with a multiwavelength measurement system (Fluostar Omeg, USA). Each measurement was taken in triplicate.

Sheep red blood cells (SRBCs) were used to detect hemolytic activity according to a previous method. Briefly, fresh SRBCs were washed with PBS, and a suspension of 10% SRBCs was mixed with solutions of Tsap or melittin at various concentrations. After incubation at 37°C for 6 h, the cells were removed by centrifugation at 800 × *g* for 10 min, and the absorbance of the supernatant at 540 nm was measured. Culture supernatant from cells that had been stimulated with 1% Triton X-100 was used as a positive control, and culture supernatant from cells that had been incubated with PBS was used as a negative control. Each measurement was taken in triplicate.

### Structured illumination microscopy (SIM) assay.

The location of Tsap AMPs in bacteria was visualized by SIM. Bacteria were cultured overnight, grown to log phase, washed three times with PBS, and coincubated with FITC-labeled AMP at a concentration of 0.5× MBC for 30 min. After being washed three times with PBS, the bacteria were stained with 4′,6-diamidino-2-phenylindole (DAPI; Sigma) for 30 min at 37°C. The cells were then washed three times with PBS, and colocalization of Tsap and bacteria was observed by SIM (Nihonika, Japan).

### Determination of ROS content.

E. coli and S. aureus were incubated overnight, transferred, grown to an OD_600_ of approximately 0.5, washed three times with PBS, adjusted to an OD_600_ of 1.0, incubated with various concentrations of AMPs and bacteria at 37°C for 1 h, and washed three times with PBS. The cell precipitate was collected and resuspended in PBS containing H_2_DCFDA. A total of 200 μL of cell suspension was added to black 96-well plates, and a microplate reader with a multiwavelength measurement system (PE, USA) was used at an excitation wavelength of 488 nm and an emission wavelength (λ) of 507 nm. Each measurement was taken in triplicate.

### Membrane potential measurements.

Changes in membrane potential after exposure to peptides were measured using DIOC_2_(3) as described previously with slight modifications (41). Specifically, after an overnight incubation of the bacteria, the bacteria were transferred to fresh medium and allowed to grow to mid-log phase. They were then collected by centrifugation at 5,000 rpm for 10 min, washed three times with PBS, adjusted to an OD_600_ of approximately 1.0, and incubated with various concentrations of AMPs or 5 μm CCCP for 1 h. After three washes with PBS, the cells were added to PBS containing DIOC_2_(3) and incubated for 30 min at 37°C. After three more washes with PBS, the numbers of red (PE) and green fluorescent (FITC) bacteria were analyzed by flow cytometry (BD, USA). More than 10,000 bacteria were analyzed in each experiment. The results are expressed as the numbers of green and red fluorescent bacteria obtained at different concentrations of AMPs. The CCCP-treated group was used as the positive control.

### Measurement of ATP content.

Total and extracellular ATP content was measured using an ATP assay kit (Beyotime, China). The protocol was performed according to the manufacturer’s instructions (42). Briefly, bacteria were incubated overnight, transferred to fresh medium, and allowed to grow to mid-logarithmic phase. They were then washed three times with PBS and adjusted to an OD_600_ of 1.0. After exposure to various concentrations of AMPs at 37°C for 1 h, the supernatants and the precipitates of the cultures were collected separately. The precipitates were digested to produce a cell lysate, which was then added to an ATP detection reagent. A total of 200 μL of cell suspension was added to black 96-well plates, and a microplate reader with a multiwavelength measurement system (PE, USA) was used at an excitation wavelength of 488 nm and an emission wavelength (λ) of 507 nm. Each measurement was taken in triplicate.

### SEM assay.

Bacteria were cultured overnight at 37°C in LB medium and grown to logarithmic cell phase after being recultured. The bacteria were washed three times with PBS and treated with Tsap-1 at 16 μg/mL and 32 μg/mL for 30 min or 1 h. The culture was then centrifuged at 4,000 × *g* for 10 min. After three washes with PBS, the cells were fixed for 2 h at room temperature in electron microscope fixative and then fixed overnight at 4°C. The cells were then dehydrated, sprayed with gold, and visualized by scanning electron microscopy (Regulus 8100; Hitachi, Japan).

### Isothermal titration calorimetry (ITC).

The binding of Tsap AMP to LPS/LTA was measured by ITC at 25°C using a Nano ITC instrument. AMP (0.1 mM/mL), LPS (1 mM), and LTA (1 mM) were dissolved in PBS. A total of 300 μL of the AMP solution was added to the bacterial culture together with either LPS or LTA (volume, 50 μL). The samples were aspirated and titrated 20 times (2.5 μL each time), and data were collected. The Tsap solution was titrated with PBS to correct for the heat of dilution. The data were analyzed using NanoAnalyze software, and binding was determined using an independent binding model. All ITC experiments were performed in triplicate.

### LPS/LTA competitive bactericidal assay.

The LPS/LTA competitive bactericidal activity of the peptides was evaluated using the experimental protocol described previously (28). S. aureus ATCCw25923 was used as a target for competitive bactericidal activity. Peptides were added to bacterial cultures containing 1 mg/mL LPS/LTA to final concentrations of 0.5× MIC, 1× MIC, 2× MIC, 4× MIC, and 8× MIC, and the cultures were incubated for 2 h. Then 10-fold serial dilutions were prepared, and CFUs were counted. The experiment was repeated independently three times.

### Endotoxin neutralization experiments.

A standard curve was established according to the instructions provided with the kit (Genscript, China). A total of 100 μL of peptides at various concentrations was added to endotoxin-free tubes, followed by the addition of 100 μL of horseshoe crab reagent, and the mixture was incubated for 30 min at 37°C with protection from light. Then, 100 μL of color development solution was added, the tubes were incubated for an additional 15 min, and the absorbance at 545 nm was measured.

### Detection of inflammation *in vivo* and *in vitro*.

HeLa cells (2.5 × 10^5^) were inoculated into 24-well plates and incubated until the cells covered the bottom of the plate. LTA (200 ng/mL) and various concentrations of Tsap peptide were then added, and the cultures were incubated overnight in a CO_2_ incubator at 37°C. The cell culture supernatant was then removed, centrifuged at 1,000 × *g* for 10 min at 4°C, and TNF-α and IL-6 concentrations were measured by human TNF-α and IL-6 enzyme-linked immunosorbent assay (ELISA) kits (Ruixin Biotech, China).

### Animal experiments.

The animal experiments described in this paper were conducted in accordance with the standards and specifications for animal experiments of Huazhong Agricultural University, with ethical approval number HAZUMO-2020-0013. All procedures complied with animal welfare and protection regulations.

The LTA-induced mice model was used to detect inflammation *in vivo*. Fifteen BALB/c female mice weighing 18 ± 2 g each were divided randomly into three groups. The mice in one of the groups were given 20 mg/kg of body weight of LTA by intranasal administration, and those in the second group were given 20 mg/kg LTA and 20 mg/kg Tsap-1 by intranasal administration. The mice in the last group were given equal amounts of PBS administered in the same way. The lung tissue of the animals was then fixed in an electron microscope fixative and sectioned for microscopic observation, and images were obtained. All the pathological images were obtained through the same microscope (Olympus, Japan), and any potential observed lines are likely the result of an artifact in the Illustrator software (Adobe, USA) or microscope.

To determine the drug protection rate fraction, S. aureus USA200 that had been cultured overnight was cultured in LB broth to an OD_600_ of 0.5, washed three times, and resuspended in PBS. Forty female BALB/c mice (18 ± 2 g each) were divided randomly into four groups. All four groups (*n* = 10) of mice were injected intraperitoneally with 1 × 10^7^ bacteria. Six hours after infection, the animals in the four groups received 20 mg/kg Tsap-1, 20 mg/kg ciprofloxacin, 10 mg/kg Tsap-1, and 10 mg/kg ciprofloxacin or an equal amount of PBS by intraperitoneal injection. The survival rate of the mice was recorded after 7 days.

For the mouse tissue load experiment, the bacteria were manipulated as described previously. Twenty female BALB/c mice (18 ± 2 g each) were divided randomly into four groups of five mice each. The animals in the four groups were injected intraperitoneally with 20 mg/kg Tsap-1 AMP, 20 mg/kg ciprofloxacin, 10 mg/kg Tsap-1, and 10 mg/kg ciprofloxacin or an equal amount of PBS. Twelve hours later, the mice were anesthetized with ether and perfused with PBS, and their livers, spleens, lungs, and kidneys were harvested. The tissues were then homogenized using a tissue grinder. Then, 10× serial dilutions of the homogenates were prepared and plated onto LB plates, and the CFUs were counted.

### Statistical analysis.

The above data were analyzed using GraphPad Prism 7.0. Student’s *t* test was used to compare the means between two specific groups, and analysis of variance (ANOVA) was applied to compare the means of more than two groups (*, *P* < 0.05; **, *P* < 0.01; ***, *P* < 0.001; ns, not significant).

### Data availability.

The data presented in this study are available on request from the corresponding author.

## Supplementary Material

Reviewer comments

## References

[1] Clancy CJ, Nguyen MH. 2022. Management of highly resistant Gram-negative infections in the intensive care unit in the era of novel antibiotics. Infect Dis Clin North Am 36:791–823. doi:10.1016/j.idc.2022.08.004. 36328637

[2] Bassetti M, Vena A, Labate L, Giacobbe DR. 2022. Empirical antibiotic therapy for difficult-to-treat Gram-negative infections: when, how, and how long? Curr Opin Infect Dis 35:568–574. doi:10.1097/QCO.0000000000000884. 36206149

[3] Nazarov PA. 2022. MDR pumps as crossroads of resistance: antibiotics and bacteriophages. Antibiotics 11:734. doi:10.3390/antibiotics11060734. 35740141 PMC9220107

[4] Liu Y-Y, Wang Y, Walsh TR, Yi L-X, Zhang R, Spencer J, Doi Y, Tian G, Dong B, Huang X, Yu L-F, Gu D, Ren H, Chen X, Lv L, He D, Zhou H, Liang Z, Liu J-H, Shen J. 2016. Emergence of plasmid-mediated colistin resistance mechanism MCR-1 in animals and human beings in China: a microbiological and molecular biological study. Lancet Infect Dis 16:161–168. doi:10.1016/S1473-3099(15)00424-7. 26603172

[5] Kanj SS, Bassetti M, Kiratisin P, Rodrigues C, Villegas MV, Yu Y, van Duin D. 2022. Clinical data from studies involving novel antibiotics to treat multidrug-resistant Gram-negative bacterial infections. Int J Antimicrob Agents 60:106633. doi:10.1016/j.ijantimicag.2022.106633. 35787918

[6] Dini I, De Biasi M-G, Mancusi A. 2022. An overview of the potentialities of antimicrobial peptides derived from natural sources. Antibiotics 11:1483. doi:10.3390/antibiotics11111483. 36358138 PMC9686932

[7] Gnanasekar S, Kasi G, He X, Zhang K, Xu L, Kang E-T. 2023. Recent advances in engineered polymeric materials for efficient photodynamic inactivation of bacterial pathogens. Bioact Mater 21:157–174. doi:10.1016/j.bioactmat.2022.08.011. 36093325 PMC9421094

[8] Ribeiro M, Sousa CA, Simões M. 2022. Harnessing microbial iron chelators to develop innovative therapeutic agents. J Advanced Res 39:89–101. doi:10.1016/j.jare.2021.10.010. PMC9263657 35777919

[9] Shen M, Forghani F, Kong X, Liu D, Ye X, Chen S, Ding T. 2020. Antibacterial applications of metal-organic frameworks and their composites. Compr Rev Food Sci Food Saf 19:1397–1419. doi:10.1111/1541-4337.12515. 33337086

[10] Huang W, Meng L, Chen Y, Dong Z, Peng Q. 2022. Bacterial outer membrane vesicles as potential biological nanomaterials for antibacterial therapy. Acta Biomater 140:102–115. doi:10.1016/j.actbio.2021.12.005. 34896632

[11] Zhu W, Wang Y, Cao W, Cao S, Zhang J. 2018. In vitro evaluation of antimicrobial combinations against imipenem-resistant Acinetobacter baumannii of different MICs. J Infect Public Health 11:856–860. doi:10.1016/j.jiph.2018.07.006. 30057349

[12] Chen J, Li Y, Wang S, Zhang H, Du Y, Wu Q, Wang H. 2022. Targeting Clostridioides difficile: new uses for old drugs. Drug Discov Today 27:1862–1873. doi:10.1016/j.drudis.2022.03.021. 35390545

[13] Barbarossa A, Rosato A, Corbo F, Clodoveo ML, Fracchiolla G, Carrieri A, Carocci A. 2022. Non-antibiotic drug repositioning as an alternative antimicrobial approach. Antibiotics 11:816. doi:10.3390/antibiotics11060816. 35740222 PMC9220406

[14] Le VVH, Davies IG, Moon CD, Wheeler D, Biggs PJ, Rakonjac J. 2019. Novel 5-nitrofuran-activating reductase in Escherichia coli. Antimicrob Agents Chemother 63. doi:10.1128/AAC.00868-19. PMC6811407 31481448

[15] Mela I, Vallejo-Ramirez PP, Makarchuk S, Christie G, Bailey D, Henderson RM, Sugiyama H, Endo M, Kaminski CF. 2020. DNA nanostructures for targeted antimicrobial delivery. Angew Chemie Int Ed Engl 59:12698–12702. doi:10.1002/anie.202002740. PMC7496991 32297692

[16] Chang RYK, Nang SC, Chan H-K, Li J. 2022. Novel antimicrobial agents for combating antibiotic-resistant bacteria. Adv Drug Deliv Rev 187:114378. doi:10.1016/j.addr.2022.114378. 35671882

[17] Zhu Z, Antenucci F, Villumsen KR, Bojesen AM. 2021. Bacterial outer membrane vesicles as a versatile tool in vaccine research and the fight against antimicrobial resistance. mBio 12:e0170721. doi:10.1128/mBio.01707-21. 34372691 PMC8406158

[18] Clark-Curtiss JE, Curtiss R. 2018. Vaccines: conduits for protective antigens. J Immunol 200:39–48. doi:10.4049/jimmunol.1600608. 29255088

[19] Jeffreys S, Chambers JP, Yu J-J, Hung C-Y, Forsthuber T, Arulanandam BP. 2022. Insights into protective immunity. Front Immunol 13:1070424. doi:10.3389/fimmu.2022.1070424. 36466845 PMC9716351

[20] Rincón-Cortés CA, Bayona-Rojas MA, Reyes-Montaño EA, Vega-Castro NA. 2022. Antimicrobial activity developed by scorpion venoms and its peptide component. Toxins 14:740. doi:10.3390/toxins14110740. 36355990 PMC9693228

[21] Wang G, Li X, Wang Z. 2016. APD3: the antimicrobial peptide database as a tool for research and education. Nucleic Acids Res 44:D1087–D1093. doi:10.1093/nar/gkv1278. 26602694 PMC4702905

[22] Cianfanelli FR, Monlezun L, Coulthurst SJ. 2016. Aim, load, fire: the type VI secretion system, a bacterial nanoweapon. Trends Microbiol 24:51–62. doi:10.1016/j.tim.2015.10.005. 26549582

[23] Tang L, Yue S, Li G-Y, Li J, Wang X-R, Li S-F, Mo Z-L. 2016. Expression, secretion and bactericidal activity of type VI secretion system in Vibrio anguillarum. Arch Microbiol 198:751–760. doi:10.1007/s00203-016-1236-2. 27172981

[24] Liu Y, Wang J, Zhang Z, Wang F, Gong Y, Sheng D-H, Li Y-Z. 2021. Two PAAR proteins with different C-terminal extended domains have distinct ecological functions in Myxococcus xanthus. Appl Environ Microbiol 87:e00080-21. doi:10.1128/AEM.00080-21. 33608292 PMC8091009

[25] Luna B, Trebosc V, Lee B, Bakowski M, Ulhaq A, Yan J, Lu P, Cheng J, Nielsen T, Lim J, Ketphan W, Eoh H, McNamara C, Skandalis N, She R, Kemmer C, Lociuro S, Dale GE, Spellberg B. 2020. A nutrient-limited screen unmasks rifabutin hyperactivity for extensively drug-resistant Acinetobacter baumannii. Nat Microbiol 5:1134–1143. doi:10.1038/s41564-020-0737-6. 32514072 PMC7483275

[26] Kaila VRI, Wikström M. 2021. Architecture of bacterial respiratory chains. Nat Rev Microbiol 19:319–330. doi:10.1038/s41579-020-00486-4. 33437024

[27] Oyinloye BE, Adenowo AF, Kappo AP. 2015. Reactive oxygen species, apoptosis, antimicrobial peptides and human inflammatory diseases. Pharmaceuticals (Basel) 8:151–175. doi:10.3390/ph8020151. 25850012 PMC4491653

[28] Zhong C, Zhang F, Zhu N, Zhu Y, Yao J, Gou S, Xie J, Ni J. 2021. Ultra-short lipopeptides against gram-positive bacteria while alleviating antimicrobial resistance. Eur J Med Chem 212:113138. doi:10.1016/j.ejmech.2020.113138. 33422980

[29] Murphey ED, Fang G, Sherwood ER. 2008. Endotoxin pretreatment improves bacterial clearance and decreases mortality in mice challenged with Staphylococcus aureus. Shock 29:512–518. doi:10.1097/shk.0b013e318150776f. 17724430

[30] Nau R, Eiffert H. 2002. Modulation of release of proinflammatory bacterial compounds by antibacterials: potential impact on course of inflammation and outcome in sepsis and meningitis. Clin Microbiol Rev 15:95–110. doi:10.1128/CMR.15.1.95-110.2002. 11781269 PMC118062

[31] Nwabuife JC, Omolo CA, Govender T. 2022. Nano delivery systems to the rescue of ciprofloxacin against resistant bacteria “E. coli; P aeruginosa; Saureus; and MRSA” and their infections. J Control Release 349:338–353. doi:10.1016/j.jconrel.2022.07.003. 35820538

[32] Bin Hafeez A, Jiang X, Bergen PJ, Zhu Y. 2021. Antimicrobial peptides: an update on classifications and databases. Int J Mol Sci 22:11691. doi:10.3390/ijms222111691. 34769122 PMC8583803

[33] Zhang Q-Y, Yan Z-B, Meng Y-M, Hong X-Y, Shao G, Ma J-J, Cheng X-R, Liu J, Kang J, Fu C-Y. 2021. Antimicrobial peptides: mechanism of action, activity and clinical potential. Mil Med Res 8:48. doi:10.1186/s40779-021-00343-2. 34496967 PMC8425997

[34] Su S-C, Hua K-F, Lee H, Chao LK, Tan S-K, Lee H, Yang S-F, Hsu H-Y. 2006. LTA and LPS mediated activation of protein kinases in the regulation of inflammatory cytokines expression in macrophages. Clin Chim Acta 374:106–115. doi:10.1016/j.cca.2006.05.045. 16899235

[35] Pogue JM, Bonomo RA, Kaye KS. 2019. Ceftazidime/avibactam, meropenem/vaborbactam, or both? Clinical and formulary considerations. Clin Infect Dis 68:519–524. doi:10.1093/cid/ciy576. 30020449

[36] Jiang Y, Chen B, Duan C, Sun B, Yang J, Yang S. 2015. Multigene editing in the Escherichia coli genome via the CRISPR-Cas9 system. Appl Environ Microbiol 81:2506–2514. doi:10.1128/AEM.04023-14. 25636838 PMC4357945

[37] Storey D, McNally A, Åstrand M, Sa-Pessoa Graca Santos J, Rodriguez-Escudero I, Elmore B, Palacios L, Marshall H, Hobley L, Molina M, Cid VJ, Salminen TA, Bengoechea JA. 2020. Klebsiella pneumoniae type VI secretion system-mediated microbial competition is PhoPQ controlled and reactive oxygen species dependent. PLoS Pathog 16:e1007969. doi:10.1371/journal.ppat.1007969. 32191774 PMC7108748

[38] Teng C-H, Xie Y, Shin S, Di Cello F, Paul-Satyaseela M, Cai M, Kim KS. 2006. Effects of ompA deletion on expression of type 1 fimbriae in Escherichia coli K1 strain RS218 and on the association of E. coli with human brain microvascular endothelial cells. Infect Immun 74:5609–5616. doi:10.1128/IAI.00321-06. 16988236 PMC1594875

[39] Roy S, Zhu Y, Ma J, Roy AC, Zhang Y, Zhong X, Pan Z, Yao H. 2019. Role of ClpX and ClpP in Streptococcus suis serotype 2 stress tolerance and virulence. Microbiological Res 223–225:99–109. doi:10.1016/j.micres.2019.04.003. 31178057

[40] Falciani C, Zevolini F, Brunetti J, Riolo G, Gracia R, Marradi M, Loinaz I, Ziemann C, Cossío U, Llop J, Bracci L, Pini A. 2020. Antimicrobial peptide-loaded nanoparticles as inhalation therapy for infections. Int J Nanomedicine 15:1117–1128. doi:10.2147/IJN.S218966. 32110011 PMC7034994

[41] Marcelletti JF, Sikic BI, Cripe LD, Paietta E. 2019. Evidence of a role for functional heterogeneity in multidrug resistance transporters in clinical trials of P-glycoprotein modulation in acute myeloid leukemia. Cytometry B Clin Cytom 96:57–66. doi:10.1002/cyto.b.21737. 30334334 PMC6340737

[42] Li N, Zhang C-X, Wang X-X, Zhang L, Ma X, Zhou J, Ju R-J, Li X-Y, Zhao W-Y, Lu W-L. 2013. Development of targeting lonidamine liposomes that circumvent drug-resistant cancer by acting on mitochondrial signaling pathways. Biomaterials 34:3366–3380. doi:10.1016/j.biomaterials.2013.01.055. 23410681

