# Supplementary material for: Effectors of the Type VI Secretion System Have the Potential to Be Modified into Antimicrobial Peptides
Source: Microbiol Spectr. 2023 Jul 20;11(4):e00308-23. doi: 10.1128/spectrum.00308-23 (PMC10434152; doi:10.1128/spectrum.00308-23)
Supplement: Supplemental file 1 — Table S1. Download spectrum.00308-23-s0001.doc, DOC file, 0.04 MB [file spectrum.00308-23-s0001.doc]

**Table S1. Strain and plasmid information**

| **Strain or Plasmid** | | **Description** | **Source or reference** |
| --- | --- | --- | --- |
| RS218 | | *ExPEC,* Wild type (WT), Human origin, O18 | Lab stock |
| Δtsap | | *ExPEC,* Mutant with gene deleted in *RS218* | This study |
| Δclpv | | *ExPEC,* Mutant with gene deleted in *RS218* | This study |
| W3110 | | *E.coil,* F-, lambda- IN (rrnD-rrnE)1 rph-1, Km^S^ | Lab stock |
| RS218/p-1835_1-324_ | | *ExPEC, WT* strain carrying the *PHSG396-1835_1-324_* overexpression vector | This study |
| RS218/p-1835_1041-1437_ | | *ExPEC, WT* strain carrying the *PHSG396-1835_1041-1437_* overexpression vector | This study |
| RS218/p-1835_1-1041_ | | *ExPEC, WT* strain carrying the *PHSG396-1835_1-1041_* overexpression vector | This study |
| RS218/p-1835_1-1437_ | | *ExPEC, WT* strain carrying the *PHSG396-1835_1-1437_* overexpression vector | This study |
| RS218/p-1835_324-1437_ | | *ExPEC, WT* strain carrying the *PHSG396-1835_1-324_* overexpression vector | This study |
| RS218/p-empty | | *ExPEC, WT strain carrying the PHSG396-18351-324 overexpression vector* | This study |
| *E. coli* ATCC 25922 | | *Standard E. coli strain* | Lab stock |
| *S. aureus* ATCC 25923 | | Standard *S. aureus strain* | Lab stock |
| *S. aureus* USA300 | | Multidrug-resistant *S. aureus* strain | Lab stock |
| S. aureus 1802043 | | Multidrug-resistant *S. aureus* strain | Lab stock |
| *S. aureus* USA200 | | Multidrug-resistant *S. aureus* strain | Lab stock |
| *S. aureus* ATCC 43300 | | Multidrug-resistant *S. aureus* strain | Lab stock |
| *Streptococcus* SC19 | | Multidrug-resistant *Streptococcus* strain | Lab stock |
| *Bacillus subtilis NCD-2* | | Multidrug-resistant *Bacillus subtilis* strain | Lab stock |
| PCN033 | | Multidrug-resistant *ExPEC* | Lab stock |
| BL21 (DE3) | | *E.coil,* F_ ompT hsdSB (rB_mB) dcm gal (DE3) | Transgene  Biotech |
| ***Plasmids*** |  | |  |
| *Pcas* | | repA101(Ts) kan Pcas-cas9 ParaB-Red lacIq Ptrc-sgRNA-pMB1 | Addgene |
| *pTargetF* | | pMB1 aadA | Addgene |
| *PHSG396* | | pUC-type bacterial cloning vector carrying a chloramphenicol-resistance gene. The MCS is similar but reversed in pHSG398. | Lab stock |
| *p-1835_1-324_* | | PHSG396 carrying gene 1835 1-324 bp region | This study |
| *p-1835_1041-1437_* | | PHSG396 carrying gene 1835 1041-1437 bp region | This study |
| *p-1835_1-1041_* | | PHSG396 carrying gene 1835 1-1041 bp region | This study |
| *p-1835_1-1437_* | | PHSG396 carrying gene 1835 1-1437 bp region | This study |
| *p-1835_324-1041_* | | PHSG396 carrying gene 1835 324-1041 bp region | This study |
| *p-1835_324-1437_* | | PHSG396 carrying gene 1835 324-1437 bp region | This study |
